# Arsenic, vinyl chloride, viral hepatitis, and hepatic angiosarcoma: A hospital-based study and review of literature in Taiwan

**DOI:** 10.1186/1471-230X-11-142

**Published:** 2011-12-26

**Authors:** Neng-Chyan Huang, Shue-Ren Wann, Hong-Tai Chang, Shoa-Lin Lin, Jyh-Seng Wang, How-Ran Guo

**Affiliations:** 1 Department of Emergency Medicine, Kaohsiung Veterans General Hospital, 386 Ta-Chung 1st Road, Kaohsiung 81362, Taiwan; 2 National Yang-Ming University, School of Medicine, 155 Linong Street Section.2, Taipei 112, Taiwan; 3 Intensive Care Unit, Department of Internal Medicine, Kaohsiung Veterans General Hospital, 386 Ta-Chung 1st Road, Kaohsiung 81362, Taiwan; 4 Department of Pathology, Kaohsiung Veterans General Hospital, 386 Ta-Chung 1st Road, Kaohsiung 81362, Taiwan; 5 Department of Environmental and Occupational Health, College of Medicine, National Cheng Kung University, 138 Sheng-Li Road, Tainan 70428, Taiwan; 6 Department of Occupational and Environmental Medicine, National Cheng Kung University Hospital, 138 Sheng-Li Road, Tainan 70428, Taiwan; 7 Sustainable Environment Research Center, National Cheng Kung University, 500 An-Ming Road Section 3, Tainan 70955, Taiwan

**Keywords:** hepatic angiosracoma, vinyl chloride, arsenic, viral hepatitis

## Abstract

**Background:**

Hepatic angiosarcoma (HAS) is a rare type of liver cancer that is often fatal, and arsenic and vinyl chloride monomer (VCM) are two major causal agents. Whereas Taiwan is an endemic area of liver cancer, epidemiologic data on HAS are limited. We reviewed the cases observed at a teaching hospital to evaluate the roles of VCM, arsenic, and viral hepatitis in the occurrence of HAS.

**Methods:**

We reviewed the medical records of patients with pathological proof of HAS from January 2000 to August 2010 at a teaching hospital which is adjacent to the major VCM processing area in Taiwan and nearby an endemic area of arsenic exposure from drinking water. We also conducted a literature review and included all patients of HAS reported in Taiwan.

**Results:**

Six male and three female cases aged from 56 to 83 years (64.6 ± 8.2 years) were identified at the hospital. The differences in clinical features between men and women were not statistically significant. None of them had exposure to VCM or arsenic in drinking water. Two had evidence of hepatitis C infection, but none had evidence of hepatitis B infection. Five male and four female cases aged 30 to 82 years (58.6 ± 15.5 years) were identified in the literature, including two with arsenic exposure and one with chronic hepatitis B infection.

**Conclusions:**

HAS is rare in Taiwan, and we found no evidence supporting a major role of VCM, arsenic in drinking water, or viral hepatitis in its occurrence.

## Background

Hepatic angiosarcoma (HAS) is a rare type of liver cancer, and some studies showed that it is related to arsenic, vinyl chloride monomer (VCM), thorium dioxide (Thorotrast), and radium [1-4]. HAS accounts for only 2% of hepatic malignancy in west countries [1,5,6], but epidemiological data in Asia are very limited, although liver cancer is relatively prevalent in this area. Many cases of HAS are asymptomatic when they are diagnosed accidently, and the most common initial complaints are nonspecific symptoms such as right upper quarterant pain, fatigue, weakness, and weight loss [1], making it rarely diagnosed antemortem [1,7].

HAS is of special interest to occupational medicine due to its correlations to vinyl chloride monomer (VCM) and other industrial chemical exposures such as arsenic pesticides in vineyard workers [7]. It is also related to arsenic exposures from other sources such as Fowler's solution to treatment of certain diseases [8,9] and drinking water containing high level of arsenic [3,10]. In some cases, HAS was reported in patient under hemodialysis (HD) [3,4] or with congestive heart failure [11].

Taiwan (Republic of China; R.O.C.) has been a major polyvinyl chloride (PVC) producer in the world for decades and has several endemic areas of arsenic exposure from drinking water. In addition, it is an endemic area of liver cancer due to the high prevalence of viral hepatitis. However, there are limited reports about HAS in Taiwan. We conducted a study to evaluate the roles of VCM, arsenic, and viral hepatitis in the occurrence of HAS in Taiwan.

## Methods

### Identification of patients at a teaching hospital

This study was conducted at Kaohsiung Veterans General Hospital in Kaohsiung in collaboration with the National Cheng Kung University in Tainan. We reviewed the medical records of patients who were diagnosed as having HAS through pathological examination of specimens obtained by fine-needle aspiration (FNA) or surgical procedures from January 2000 to August 2010. This 1330-bed hospital is adjacent to the major PVC production area in Taiwan and nearby an endemic area of arsenic exposure from drinking water generally known as the blackfoot diseases endemic area [3]. From each patient, we collected data on demographic characteristics, medical history, hemogram, biochemistry, markers of hepatitis B (HBV) and C (HCV) virus infections, markers associated with hepatic tumor, and clinical course. In addition, we collected information of serial image studies using sonography, magnetic resonance imaging (MRI), and computed tomography (CT), which included tumor size and location. We also collected information on occupational history and environmental exposures, including underground water drinking. The study protocol was approved by the Human Research Committee of the hospital.

### Identification of cases in the literature

In order to identify cases of HAS reported in Taiwan, we also conducted a thorough literature search in the PubMed database using "angiosracoma" as the key word and review the articles retrieved. Further search of literature was conducted through reviewing the references listed in retrieved articles.

### Statistical analyses

We performed statistical analyses using SPSS software for Windows (Version 12; SPSS Inc., Chicago, IL, USA). All data were expressed as mean ± standard deviation, and the differences between two groups were evaluated using the Mann-Whitney U test for continuous variables and Fisher's exact test for categorical variables. A *p *value of less than 0.05 was considered to be statistically significant.

## Results

**Figure 1:**
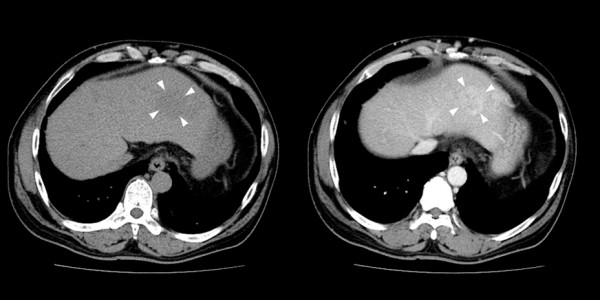
**The abdominal computed tomograph in a patient of hepatic angiosarcoma (Case 9)**. A low-density mass (3.5 cm) with persistent contrast enhancement (29/54/68 hu) in left hepatic lobe is noted (arrowhead) (left: non-contrast phase, right: venous phase). Several enhancing lesions (1-3 cm) in right lobe of liver S5 are not well demonstrated in the venous phase (not shown in figure). Irregular liver surface, splenomegaly, and collateral circulation are noted, indicating cirrhosis.

**Figure 2:**
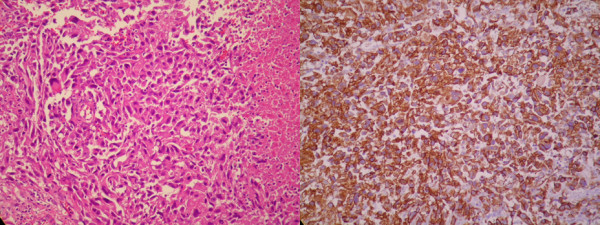
**The pathological features of hepatic angiosarcoma (Case 9)**. The sections of the specimen show a picture of liver tissue with sheets of spindle tumor cells with focal necrosis (Left, H&E stain, x200). Focal vascular channels are also lined by atypical cells. The tumor cells show positive staining in the CD31 (Right, x200), CD34, factor VIII, D2-40 and c-kit stains, and negative for AE1/AE3, hepatocyte, S100, and HHF-35 stains.

Nine patients (six men, three women) of HAS were identified at the hospital during the study period (Table 1). The average duration of follow-up was 14 months, and two patients died within 3 months (Table 1). Eight of the nine patients had bilateral lobes involvement, and all had multiple segments invasion. In some patients, abdominal CT revealed contrast enhanced lesions persistently, while in the others it showed hypo-attenuated lesions in the venous phase (Figure 1). The diagnosis was made on the basis of FNA in five patients and surgery in four. Histological examination showed focal vascular channels lined by atypical cells with positive staining for at least one of the following markers: CD31, CD 34, or factor VIII (Table 2) (Figure 2). The age at diagnosis ranged from 56 to 83 years (64.6 ± 8.2 years), and it was similar between men (64.8 ± 10.0 year) and women (64.0 ± 4.6 year) (*p *= 0.80) (Table 2). None of the differences in other characteristics reached statistical significance.

**Table 1:** Clinical features of patients of hepatic angiomyosarcoma

Case	Sex	Age (year)	Chief Complaint	Comobidity	Alcohol Drinking	Hepatitis	Treatment	Metastasis	Follow-up	Immunostaining
1	M	65	liver tumor	DM	yes	none	C	none	3 M	CD31, Factor VIII
2	M	62	abdominal pain	HTN, gout	No	none	C	none	1 W	CD31, vimentin
3	M	67	right subcostal pain	DM, HTN	No	none	OP+C/T	lung, subphrenic, peritoneum	44 M	CD31, CD34, Factor VIII, FLI-1, vimentin,
4	F	69	general weakness	DM, HTN, gastric GIST	No	none	OP+C/T+R/T	brain, spine	37 M	CD31
5	M	83	general edema	HTN, CAD, COPD	No	none	C	none	8 M	CD31
6	M	56	epigastric mass	DM, HTN, liver rupture	No	none	TAE+OP	none	4 M	CD31
7	F	60	liver tumor	hypothyroidism, uremia	No	HCV	C	none	2 M	CD34
8	F	63	right abdominal pain	liver rupture	No	none	OP+C/T	peritoneum, duodeum, colon	3 M	CD31
9	M	56	liver tumor	L/C, EV, left hip AVN	yes	HCV	TAE+HAIC	none	24 M	CD31, CD34, D2-40, c-kit, Factor VIII

DM = diabetes mellitus; HTN = hypertension; GIST = gastrointestinal stromal tumor; CAD = coronary arterial disease; COPD = chronic obstructive pulmonary disease; L/C = liver cirrhosis; EV = esophageal varies; AVN = avscular necrosis; OP = operation; TAE = transarterial embolization; C/T = chemotherapy; C = conservative treatment; HAIC = hepatic arterial infusion chemotherapy; M = month; W = week.

**Table 2:** Demographic data on patients of hepatic angiomyosarcoma

	Men	Women	*p *value
Number of patients	6 (66.7%)^a^	3 (33.3%)^a^	
Max. tumor size (cm)	8.9 ± 3.4 (3.9-13.5)	13.5 ± 7.1 (8.4-18.5)	0.51
Age (year)	64.8 ± 10.0 (56-83)	64.0 ± 4.6 (60-69)	0.80
Pathology			0.52
FNA	4	1	
Surgery	2	2	
Treatment			0.45
Operation	1	0	
OP+C/T	1	1	
OP+C/T+R/T	0	1	
TAE+HAIC	1	0	
TAE+OP	1	0	
Conservative	3	1	
Metastasis	1	2	0.23
Liver rupture	1	1	> 0.95
Hepatitis			> 0.95
HCV	1	1	
Alcohol drinking	2	0	0.50
Uremia in HD	0	1	0.33

All data of tumor size and age expressed as mean ± standard deviation; FNA = fine needle aspiration; OP = operation; C/T = chemotherapy; R/T = radiotherapy; HAIC = hepatic arterial infusion chemotherapy; TAE = transarterial embolization; HCV = hepatitis C virus; HD = hemodialysis.

^a^Data express as percentage (%) of all patients.

Two patients received transarterial embolization (TAE), including one receiving operation after the TAE due to liver rupture and the other receiving hepatic arterial infusion chemotherapy (HAIC) after the TAE. Of the four patients who received operation, three developed multiple metastases afterwards with post-operative chemotherapy, including one with radiotherapy simultaneously and another suffering from liver rupture (Table 1). Four patients received conservative treatment only. The difference in the maximum tumor size between men (8.9 ± 3.4 cm) and women (13.5 ± 7.1 cm) did not reach statistical significance (*p *= 0.51) (Table 2), and the two patients who suffered from liver rupture had the largest and the third largest tumor sizes (18.5 and 10.7 cm, respectively).

Four of the patients had diabetes mellitus, including three with hypertension, and two non-diabetic patients also had hypertension. Only one patient had a concurrent tumor, which was a gastrointestinal stromal tumor (Table 1). At diagnosis, the chief complaint included liver tumor in four patients (44.4%), abdominal pain in three (33.3%), and non-specific complaint in two (22.2%) (Table 1). The hemogram and biochemistry data revealed no differences between men and women (all *p *values > 0.05) except that white blood cell counts were higher in women (*p *= 0.02), which was attributed to one with liver rupture and another with a concurrent tumor (GIST) and metastasis (Table 3). Three diabetic men and one woman had high blood sugar levels, and the hemodialytic woman was the only patient with elevated serum creatinine and total bilirubin. Other hemogram and biochemistry data were within normal limits (Table 3). All patients had normal values of tumor markers associated with hepatic tumor, including alfa-fetoprotein (AFP), carcinoembryonic antigon (CEA), and carbohydrate antigen 19-9 (CA199).

**Table 3:** Laboratory data of patients of hepatic angiosarcoma

Variable	All (n = 9)	Men (n = 6)	Women (n = 3)	*p *value
WBC (/Cumm)	7461 ± 2785	5806 ± 1471	10770 ± 995	0.02
Hgb (g%)	12.1 ± 1.8	12.7 ± 2.0	10.8 ± 0.6	0.24
Platelet (k/Cumm)	176.2 ± 104.6	182.2 ± 73.2	164.3 ± 173.3	0.61
Blood Sugar (mg/dL)	147.3 ± 53.5	158.2 ± 63.9	125.7 ± 13.7	> 0.95
BUN (mg/dL)	14.0 ± 4.9	15.3 ± 5.3	14.4 ± 8.0	0.50
Creatinine (mg/dL)	1.7 ± 2.1	1.1 ± 0.2	3.0 ± 3.7	0.90
Sodium (mmol/L)	137.1 ± 2.5	138.0 ± 1.7	135.3 ± 3.2	0.24
Potasium (mmol/L)	3.9 ± 0.6	4.0 ± 0.4	3.8 ± 1.1	0.80
Calcium (mg/dL)	9.2 ± 0.7	9.0 ± 0.2	9.4 ± 1.4	> 0.95
T-Bilirubin (U/L)	3.4 ± 7.1	1.0 ± 0.6	10.8 ± 14.3	0.50
AST (U/L)	39.1 ± 17.9	42.3 ± 19.1	29.5 ± 13.4	0.40
ALT (U/L)	28.9 ± 17.5	32.5 ± 18.0	21.7 ± 17.4	0.44
ALP (U/L)	171.4 ± 77.4	164.0 ± 68.6	193.5 ± 130.8	0.74
rGT (U/L)	131.3 ± 23.2	120.0 ± 0.0	137.0 ± 29.7	> 0.95
CK (U/L)	47.0 ± 20.8	46.5 ± 14.8	47.3 ± 27.5	> 0.95
Amylase (U/L)	36.3 ± 8.5	38.0 ± 11.3	33.0 ± 0.0	> 0.95
Albumin (g/L)	3.5 ± 0.3	3.6 ± 0.3	3.5 ± 0.0	0.76

WBC = white cell count; Hgb = hemoglobulin; BUN = blood urea nitrogen; AST = aspartate aminotransferase; ALT = alanine aminotransferase; rGT = r-Glutamyl Transpeptidase; CK = creatine kinase.

None of the patients was a HBV carrier, but two had HCV infection, including a uremic patient under maintenance HD (Table 1). Two patients had drinking habit, and one of them was recognized as alcoholism who had liver cirrhosis (Table 1). As to occupational history, only the woman HCV carrier under maintenance HD had contact with PVC at work, which was during the manufacture of plastic products, but none of the patients had occupational exposure to VCM, radium, or arsenical pesticides. Furthermore, none of the patients recalled exposure to thorium dioxide or using Fowler's solution, and none of them have ever lived in the BFD area, where the underground water had high levels of arsenic.

We reviewed the literature on HAS in Taiwan and found nine more patients (five men, four women) of HAS, and the age at diagnosis ranged from 30 to 82 years (58.6 ± 15.5 years)[3,12-19] (Table 4). Only one patient had hepatitis B infection. The differences in the mean age at diagnosis, male-to-female ratio, and seroprevalence rates of HBV and HCV infection between our cases and cases reported in the literature did not reach statistical significance. As to occupation exposures, only one had chronic exposure of arsenic pesticide. One of the patients had diabetes mellitus, one had hypertension, and one had uremia under maintenance HD. At diagnosis, the chief complaints included abdominal pain or fullness in four patients (44.4%), liver tumor in three (33.3%), lower back pain in one (11.1%), and fever in one (11.1%) (Table 4). Three of the patients received operation and TAE, one received operation, and one received TAE. Three patients had liver rupture, including one with operation and TAE, one with operation, and one with conservative treatment. Metastasis was observed in two patients, including one in the spleen and the other in the L2 spine.

**Table 4:** Previously reported cases of hepatic angiomyosarcoma in Taiwan

Reference	Sex	Age (year)	Chief Complaint	Comobidity	Alcohol Drinking	Hepatitis	Treatment	Metastasis	Follow-up	Immunostaining
Weng et al. [12]	M	42	fever	none	no	none	TAE+OP	none	7 M	Factor VIII, UEA-I, vimentin,
Tsai et al. [13]	M	56	liver tumor	RA, OA knees, liver rupture	no	HBV	C	spleen	9 W	Factor VIII, UEA-I, vimentin, CD34
Tsai et al. [14]	M	30	epigastralgia, general weakness	None	no	none	TAE+OP	none	17 D	NA
Liang et al. [15]	F	59	liver tumor	none	no	none	OP	none	24 M	Factor VIII, vimentin
Tsai et al. [16]	M	60	dyspnea, abdominal pain	DM, liver rupture	no	none	TAE	none	24 D	NA
Hsiao [17]	F	82	abdominal fullness, abdominal pain, nausea	HTN, CRI, PUD, L/C	no	none	none	none	42 M	CD34
Ho et al. [3]	F	68	abdominal pain	uremia in HD, HTN, ureteral papilloma, liver rupture	no	none	OP+TAE+R/T	spine	42 M	Factor VIII
Hsu [18]	M	72	lower back pain	aortic aneurysm	no	none	OP	none	NA	NA
Cheng et al. [19]	F	58	liver tumor	none	no	none	OP+C/T	none	14 M	Factor VIII, vimentin

DM = diabetes mellitus; HTN = hypertension; OA = osteoarthritis; CRI = chronic renal insufficiency; PUD = peptic ulcer disease; L/C = liver cirrhosis; OP = operation; TAE = transarterial embolization; C/T = chemotherapy; R/T = readiotherapy; C = conservative treatment; M = month; W = week; D = day; NA = not available.

## Discussion

HAS is rare, but is the most common malignant mesenchymal tumor of liver [1,5,6,20-22]. A study in the New York State found an annual incidence of 0.26 per million [23], and another study estimated that the mortality rate was 0.075 cases per million population per year in the Unites States and that there were about 17 cases each year in the whole country [2]. In Western countries, it occurs more frequently in men with a male-to-female ratio of about 3:1 to 4:1, and most frequently in the sixth to seventh decades of life [1,2,7]. In Taiwan, an analysis of data from the National Cancer Registry Program from 1981 to 1999 found only 26 patients, and the male-to-female ratio was 1.9:1 [3], which is close to our finding of 2.0:1, but much lower than the 3:1 ratio in hepatic carcinoma (HCC) [24]. A study in Shanghai, People's Republic of China (PRC) [25] found 6 cases of HAS in 5,487 consecutive cases of primary liver cancers from 1991 to 2005, which was higher than the proportion of 8 in the 40,832 cases from 1980 to1999 in a study in Taiwan [24]. The Shanghai study reported a male-to-female ratio of 2.0:1, so did a study of 6 consecutive cases of HAS from 2000 to 2005 in Ganzhou, PRC [26]. The male-to-female ratio in the Chinese population appears to be quite consistent across studies and is lower than those reported in the Western countries.

While the most common presenting symptoms of HAS were reported to be abdominal pain and non-specific systemic complaints such as weakness, fatigue, weight loss, and anorexia that appear in 25-50% patients [1,7], we found that a substantial number of patients were asymptomatic cases identified during routine health examinations, highlighting the importance of periodical health check-ups. Hepatomegaly with ascites, jaundice, and splenomegaly are the most common findings on physical examination [7]. Of the 18 cases in this series, one patient identified at the hospital and one previously reported case in Taiwan had uremia, and one of them had history of HCV infection [3], which is similar to a case reported in Japan [4].

Spontaneous liver rupture is not uncommon and associates with high mortality and morbidity rates, which presents with intra-abdominal bleeding occurring in 27% of HAS patients [1]. Five patients of HAS with liver rupture, including two in our series and three in previous reports [3,13,16], had been identified in Taiwan, and two of them (one patient in our series and one previously reported case [3]) received emergent surgical intervention and survived for a period of time till another life-threatening event that cost their lives. Emergent surgery seems effective in the treatment of liver rupture.

Early reports of HAS often focus on its associations with environmental carcinogens such as VCM, arsenic in pesticide or drinking water, thorium dioxide, radium, and Fowler's solution, but exposures to these agents have become rare nowadays. Of the 135 patients reported by Falk et al., 126 (75%) were attributed to uncertain etiology [2]. Similarly, none of the nine patients in the current study could be attributed to specific etiology, and of the nine previously reported cases in Taiwan, only two were found to have chronic exposure to arsenic pesticide or arsenic in drinking water. In total, 15 of the 18 cases (83.3%) of HAS in Taiwan had no known etiology.

Although HBV infection increases the risk of liver cancer in VCM workers [26,27], none of the 18 cases had exposure to VCM, and only one previously reported patient had chronic HBV infection. Taiwan is an endemic area of viral hepatitis, including HBV and HCV infection. The reported seroprevalence of HBsAg ranged from 15% to 20% in Taiwan [28-30], and the seroprevalence of HBV we observed (5.6%; 1 in 18 cases) was lower, which does not support HBV infection being a risk factor of HAS. On the other hand, 2 of the 18 patients had HCV infection. More than 170 million people worldwide are chronically infected with the HCV, which causes over 1 million deaths resulting from cirrhosis and liver cancers [31]. The reported seroprevalence of anti-HCV has a very wide range in Taiwan [32-35] and reached 60% in some townships in the southwestern coast [34], which is higher than the prevalence we observed (11.1%, 2 in 18 cases). An association between HCV and HAS has not been confirmed, and further studies are necessary to clarify their relationship. One of our cases had uremia, and we found another case of HAS in a uremia patient with a history of HCV infection in the literature [4]. HCV infection is a significant cause of morbidity and mortality in HD patients, and the prevalence of HCV in HD population varies from 1.9 to 84.6% [36]. A study in Taiwan observed an anti-HCV seroprevalence of 33.2% in HD patients [37].

In previous studies, more than 70% of HAS patients have multifocal or multinodular lesions [21,38], and most of them have metastatic lesions at the time of presentation such as lung or splenic metastasis [1,38]. The prognosis of patients with HAS is poor with a median survival of around 6 months, and only 3% survived for more than 2 years [1,2]. In our study, three patients identified at the hospital and one previously reported case had metastasis at the time of diagnosis. Three of them received operation, and two received post operation chemotherapy. Consequently, three of our patients and three reported patients lived for more than 2 years (24, 37, and 44 months and 24, 42, and 42 months, respectively), although some survived only 3 months after diagnosis. Therefore, aggressive treatment may still prolong the survival of patients with metastases.

Our study has a few limitations. First, this was a retrospective study, which limits the amount of data to be collected. On the other hand, because all the 9 cases in the literature were collected from case reports which were also on the basis of medical records, information on the 9 cases in our series and the 9 cases in the literature should be comparable. Second, the total number of cases is small, which limits further analysis. Nonetheless, together with cases reported previously, only a small portion of the patients had exposures to arsenic or viral hepatitis, and none had exposures to VCM, even though these risk factors are relatively prevalent in Taiwan.

## Conclusions

HAS is rare in Taiwan, and we found no evidence supporting a major role of VCM, arsenic in drinking water, or viral hepatitis in its occurrence. Further studies are necessary to clarify the etiologies of HAS in Taiwan.

## Authors' contributions

NCH and HRG conceived the study, and SRW, HTC and SLL helped the design of the study. NCH, SRW, HTC and SLL participated in the patient enrolment and data collection and JSW carried out the pathological examinations. NCH and HRG analyzed the data and drafted the manuscript, and SRW, HTC, SLL, and JSW helped the revision of the manuscript. All authors read and approved the final manuscript.

## Competing interests

The authors declare that they have no competing interests.

## Pre-publication history

The pre-publication history for this paper can be accessed here:

http://www.biomedcentral.com/1471-230X/11/142/prepub
